# Fully automated artificial intelligence–based echocardiographic analysis for global longitudinal strain monitoring and cancer therapy–related cardiac dysfunction detection in breast cancer patients

**DOI:** 10.1093/ehjdh/ztag097

**Published:** 2026-06-19

**Authors:** Yoshihito Saijo, Robert Zheng, Yuichiro Okushi, Yuka Nomura, Yukina Hirata, Hiroaki Inoue, Hirotsugu Yamada, Kenya Kusunose, Masataka Sata

**Affiliations:** Department of Cardiovascular Medicine, Tokushima University Hospital, 2-50-1, Kuramoto-cho, Tokushima 7708503, Japan; Department of Cardiovascular Medicine, Tokushima University Hospital, 2-50-1, Kuramoto-cho, Tokushima 7708503, Japan; Department of Cardiovascular Medicine, Tokushima University Hospital, 2-50-1, Kuramoto-cho, Tokushima 7708503, Japan; Ultrasound Center, Tokushima University Hospital, 2-50-1, Kuramoto-cho, Tokushima, Japan; Ultrasound Center, Tokushima University Hospital, 2-50-1, Kuramoto-cho, Tokushima, Japan; Department of Thoracic, Endocrine, Surgery and Oncology, Tokushima University Hospital, 2-50-1, Kuramoto-cho, Tokushima, Japan; Department of Cardiovascular Medicine, Tokushima University Hospital, 2-50-1, Kuramoto-cho, Tokushima 7708503, Japan; Department of Cardiovascular Medicine, Nephrology and Neurology, University of the Ryukyus, Okinawa, Japan; Ultrasound Center, Tokushima University Hospital, 2-50-1, Kuramoto-cho, Tokushima, Japan

**Keywords:** CTRCD, Artificial intelligence, Breast cancer, Cardiac dysfunction, Chemotherapy, GLS

## Abstract

**Aims:**

Global longitudinal strain (GLS) is essential for the early detection of cancer therapy–related cardiac dysfunction (CTRCD). A fully automated echocardiographic analysis system using artificial intelligence (AI) may improve workflow efficiency in cardio-oncology. We sought to evaluate the feasibility and diagnostic performance of a fully automated AI-based echocardiographic system in breast cancer patients receiving cardiotoxic chemotherapy.

**Methods and Results:**

In this prospective observational study, patients with breast cancer undergoing anthracyclines and/or HER2-targeted therapy between January 2022 and June 2025 were enrolled. Transthoracic echocardiography was performed at baseline and every 12 weeks. GLS was measured manually by two experts and automatically by a fully automated AI-based analysis system. A total of 92 patients (456 echocardiographic studies) were analysed. AI-derived GLS values were significantly lower than expert measurements (17.7 ± 2.9% vs. 18.4 ± 2.8%, *P* = 0.007). Correlation and agreement between the two methods were moderate (*R* = 0.64, intraclass correlation coefficient = 0.63). On linear mixed-effects modelling, longitudinal changes in GLS were not significantly different between methods (*P* = 0.72). GLS-based CTRCD was detected in 31.5% of patients by experts and 34.8% by AI (*P* = 0.58), with similar detection timing (*P* = 0.47). Diagnostic agreement was substantial (κ = 0.68, *P* < 0.001).

**Conclusion:**

The fully automated AI-based echocardiographic system demonstrated acceptable agreement and diagnostic performance for GLS assessment and showed a similar ability to track temporal relative GLS changes and identify CTRCD. However, systemic underestimation of absolute GLS values may contribute to threshold-based classification discordance in borderline cases.

## Graphical Abstract

**Graphical Abstract:**
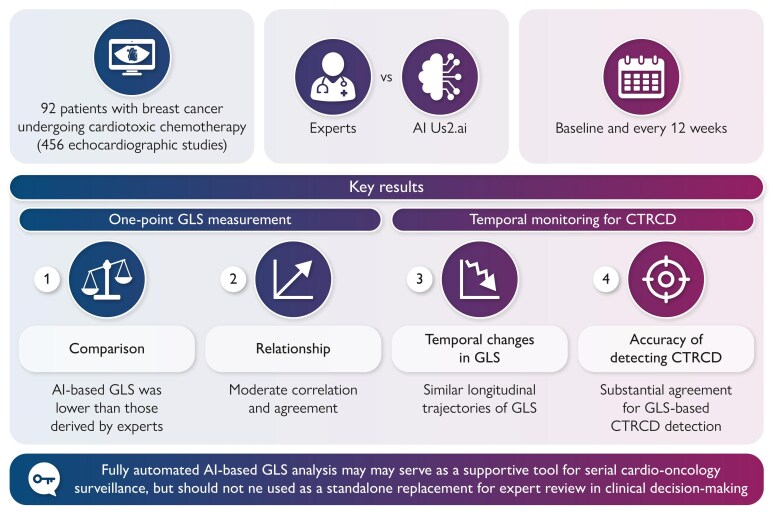
This prospective study evaluated a fully automated AI-based echocardiographic analysis system for longitudinal GLS monitoring during cardiotoxic chemotherapy. AI-derived GLS values were lower than expert measurements but demonstrated moderate correlation (*R* = 0.64) and agreement (ICC = 0.63) with experts-derived GLS. Temporal GLS trajectories were highly concordant between AI and experts, and the AI-based system showed substantial diagnostic agreement for GLS-based CTRCD detection (κ = 0.68). These findings support that fully automated AI-based GLS analysis may serve as a supportive tool for serial cardio-oncology surveillance, but should not be used as a standalone replacement for expert review in clinical decision-making.

## Introduction

Longitudinal echocardiographic surveillance is essential for the early detection of cancer therapy–related cardiac dysfunction (CTRCD) in patients undergoing chemotherapy.^1,2^ Global longitudinal strain (GLS) is particularly valuable because it detects subtle myocardial injury before a measurable decline in left ventricular ejection fraction (LVEF).^3,4^ However, the increasing demand for echocardiography, along with additional assessments such as GLS, has placed a substantial burden on both patients and healthcare workers.^5^ The routine use of GLS assessment remains limited by technical complexity, inter-observer variability, and additional examination and analysis time.^6,7^ To address these challenges, automated echocardiographic analysis systems using artificial intelligence (AI) have been developed. The Us2.ai platform enables fully automated image selection, cardiac chamber quantifications, variable measurements including GLS, and report generation.^8^ Previous studies have shown that this system can reduce examination and reporting time by up to 70%.^9^ However, despite these workflow advantages, the diagnostic utility of such an automated system, particularly the ability to reliably track temporal changes in GLS, has not yet been established. Importantly, the clinical use of GLS in cardio-oncology depends not only on cross-sectional agreement at a single time point, but also on reliable tracking of relative serial changes from baseline, because current guidelines define GLS-based CTRCD as a >15% relative reduction in GLS from baseline. This knowledge gap represents a critical unmet need in cardio-oncology, where serial GLS monitoring plays a pivotal role in CTRCD surveillance. The aim of the present study was to evaluate the feasibility and diagnostic performance of a fully automated AI-based echocardiographic analysis system in breast cancer patients receiving cardiotoxic chemotherapy, with a particular focus on its ability to track temporal changes in GLS and detect CTRCD.

## Methods

### Study population

In this prospective observational study, consecutive breast cancer patients scheduled to receive chemotherapy for breast cancer (including anthracyclines and/or HER2-targeted therapies) were prospectively enrolled between January 2022 and June 2025. Eligible patients underwent baseline transthoracic echocardiography prior to chemotherapy and follow-up studies approximately every 12 weeks for at least three examinations. Patients were excluded if they had fewer than three serial echocardiograms, inadequate image quality for manual GLS assessment, pre-existing cardiomyopathy, or moderate and severe valvular heart disease. The study was conducted in accordance with the Declaration of Helsinki and approved by the institutional Ethics Committee of Tokushima University (Institutional Review Board number: 4771). Written informed consent for participation in the study and for the use of clinical and imaging data about research purposes was obtained from all patients prior to enrolment.

### Study endpoints

The primary endpoint of the present study, pre-specified before data collection, was the diagnostic performance of the fully automated AI-based echocardiographic system compared with expert manual measurements. The primary performance targets were (1) agreement in GLS measurements, assessed by the intraclass correlation coefficient (ICC) with an exploratory benchmark of ≥0.7;^10^ and (2) concordance in detection of GLS-based CTRCD between AI and expert assessment. Secondary endpoints included the comparison of temporal GLS trajectories between AI and expert measurements, and the diagnostic accuracy for CTRCD detection expressed as sensitivity, specificity, positive predictive value, and negative predictive value.

### Echocardiographic assessment

Echocardiographic images were acquired using Vivid E9 or E95 ultrasound systems (GE Healthcare, Chicago, IL). For strain analysis, apical long-, two-, and four-chamber views were obtained over two cardiac cycles with frame rates ≥40 frames per second. GLS was manually measured by two experienced readers blinded to clinical information using the EchoPAC software version 204 (GE Healthcare, Chicago, IL). The mean GLS value of two expert measurements was used for analysis. Conventional echocardiographic measurements were reviewed and measured according to current guidelines.^11,12^

### Artificial intelligence assessment

The same echocardiographic images were analysed using the fully automated AI-based echocardiographic analysis software developed by Us2.ai (commercially available, CE-marked, and Food and Drug Administration-approved software, version 1.4.1) to obtain GLS values. Images were transferred to analysing system as digital imaging and communications in medicine files. No manual adjustment of tracing lines was performed. Echocardiographic studies were excluded from the analysis if any apical view failed to generate a valid GLS value.

### Definition of CTRCD

CTRCD was decided using LVEF and GLS according to current guidelines.^1^ GLS-based CTRCD was defined as a relative reduction in GLS by >15% from baseline, and LVEF-based CTRCD was defined as a new reduction in LVEF to <40%, a ≥10% point decrease in LVEF resulting in an LVEF of 40%–49%; or a <10% point decrease in LVEF to 40%–49% accompanied by abnormal GLS.

### Sample size calculation

The sample size was determined a priori based on a precision-based approach for the ICC between AI- and expert-derived GLS measurements.^10^ Assuming an expected ICC of 0.70, a desired 95% confidence interval (CI) half-width of 0.10, and a two-sided *α* of 0.05, 85 patients were required according to Bonett’s method.^13^ Considering repeated measurements and potential missing data, we planned to include at least 90 patients, and finally analysed 92 patients in the present study.

### Statistical analysis

Continuous variables were expressed as mean ± standard deviation for normal distribution or median and 25th to 75th interquartile range (IQR) for skewed distribution, and categorical data were expressed as number and percentage. The correlation of continuous variables between expert and AI measurements was assessed using the Pearson correlation coefficient. Differences between expert and AI measurements were assessed by paired *t*-test. The agreement of GLS measurement between expert and AI was evaluated with the ICC (2, 1) with two-way mixed model and absolute agreement,^10^ a Deming regression with error ratio = 1, and the Bland–Altman analysis with mixed-effects modelling to account for repeated measures.^14^ On the Bland–Altman analysis, the 95% limits of agreement were calculated as bias ± 1.96 times the square root of the sum of the between-subject variance and the residual variance. Temporal changes in GLS were analysed with a linear mixed-effects model including fixed effects for method (AI vs. experts), time, and their interaction. An autoregressive covariance structure was selected based on model fit and clinical plausibility. Parameter estimation was performed using restricted maximum likelihood. Detection rates of GLS-based CTRCD between AI and experts were compared using the McNemar test, and detection timing of GLS-based CTRCD was compared using the Wilcoxon signed-rank test. Diagnostic agreement for GLS-based CTRCD detection was evaluated by Cohen’s kappa coefficient. Statistical analysis was performed using SPSS version 25 (SPSS Inc., Chicago, IL) and R software version 4.5.2 (R Foundation for Statistical Computing). A *P-*value of <0.05 was considered significant.

## Results

### Study participants

**Figure 1:**
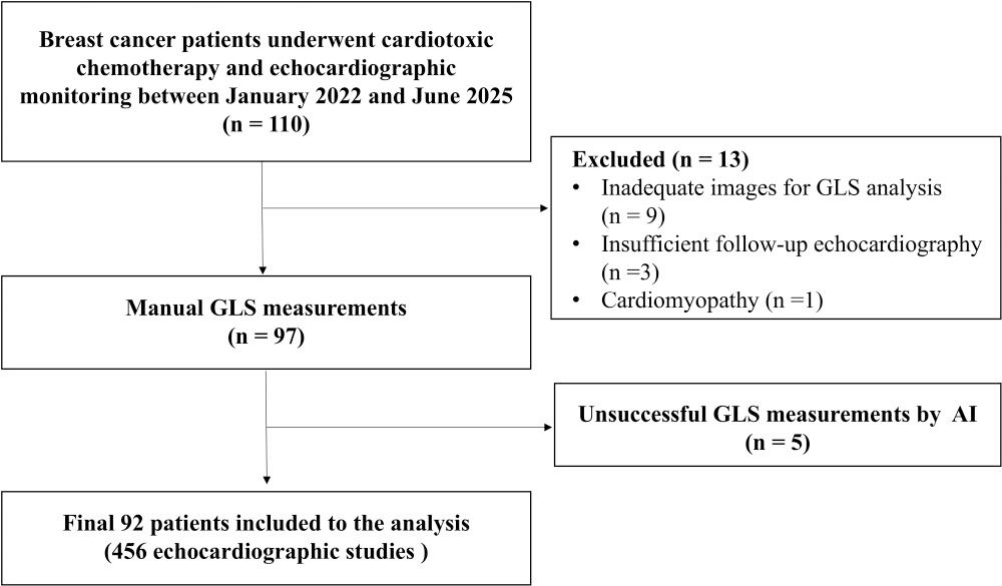
Flowchart of the study cohort. AI, artificial intelligence; GLS, global longitudinal strain.

A total of 110 patients were included in the present study. Of them, three patients were excluded due to a lack of follow-up echocardiography, nine patients due to inadequate images for manual GLS measurement, and one patient due to the presence of hypertrophic cardiomyopathy. Additionally, five patients were excluded due to unsuccessful GLS measurements by AI, and AI successfully generated GLS values in 94.8%. Finally, 92 patients with 456 echocardiographic studies (median follow-up time: 17.8 months [IQR, 15.3–18.0 months]) were included in the final analysis, as shown in *Figure 1*. The specific reasons for unsuccessful AI-derived GLS measurements could not be obtained were not available in the present study.

### Patient characteristics

Patient characteristics are shown in *Table 1*. Mean age was 57 ± 13 years, and all patients were female. Hypertension was present in 30.4% of patients, and 13.0% were on renin-angiotensin system inhibitors at baseline. According to chemotherapy, 79.3% of patients received anthracycline, 55.4% received HER2-targeted therapy, and 37.0% underwent combination therapy with both anthracyclines and HER2-targeted therapy. Mean cumulative anthracycline dose (doxorubicin equivalent dose) was 333 ± 55 mg/m^2^. Mean LVEF at baseline was 65 ± 3%. Other echocardiographic parameters are shown in *Table 2*, and no patient had moderate or severe valvular disease.

**Table 1:** Patient characteristics

	*n* = 92
**Age, y**ears	57 ± 13
**Female**	92 (100)
**Body mass index, kg/m^2^**	22.8 ± 3.7
**Hypertension**	28 (30.4)
**Diabetes mellitus**	5 (5.4)
**Cardiovascular disease**	2 (2.2)
**Current and former smoker**	13 (14.1)
**Medications at baseline**	
** Calcium-channel blocker**	19 (20.6)
** β-blocker**	4 (4.3)
** Mineral corticoid receptor**	2 (2.2)
** ACEIs/ARBs**	12 (13.0)
**Chemotherapy**	
** Anthracyclines**	73 (79.3)
** HER2-targeted therapy**	51 (55.4)
** Anthracyclines + HER2-targeted therapy**	34 (37.0)
** Cumulative anthracycline dose, mg/m^2^**	333 ± 55

Values are mean ± SD or *n* (%).

ACEIs, angiotensin-converting enzyme inhibitors; ARBs, angiotensin II receptor blockers.

**Table 2:** Echocardiographic variables at baseline

	*n* = 92
**Left ventricular end-diastolic volume, mL**	78 ± 17
**Left ventricular end-systolic volume, mL**	28 ± 7
**Left ventricular ejection fraction, %**	65 ± 3
**Left atrial volume index, mL/m^2^**	25 ± 7
**Peak aortic valve velocity, m/s**	1.3 ± 0.3
**Peak tricuspid regurgitation velocity, m/s**	2.1 ± 0.3
**E/A ratio**	1.1 ± 0.4
**E/e’**	7.8 ± 2.6

Values are mean ± SD.

### GLS measurements

**Figure 2:**
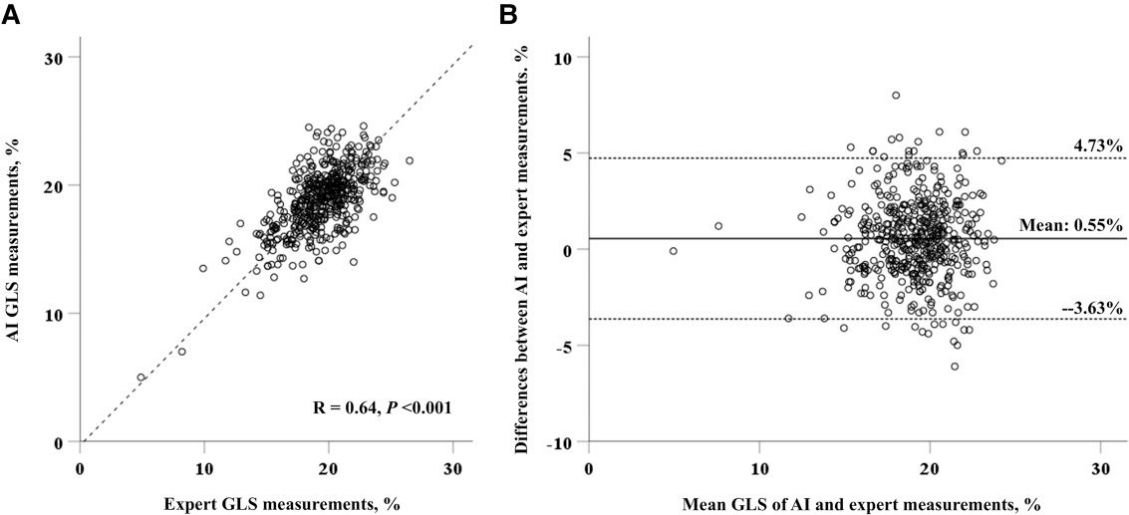
Correlation and agreement of GLS measurements between experts and AI. (*A*) Scatter plot showing the correlation between experts- and AI-derived GLS measurements, (*B*) Bland–Altman plots showing agreement between expert- and AI-derived GLS measurements. The solid line indicates the mean bias, and the dotted lines represent the upper and lower limits of agreement. Bland–Altman analysis accounting for repeated measurements within individuals showed a mean bias of 0.55%, limits of agreement from −3.63% to 4.73%, and no significant proportional bias. AI, artificial intelligence; GLS, global longitudinal strain.

A significant difference was observed in GLS measurements between AI and experts, with AI-derived GLS being significantly lower than those measured by experts (17.7 ± 2.9% vs. 18.4 ± 2.8%, *P* = 0.007). There was a moderate correlation of GLS measurements between the two methods on Pearson’s correlation coefficient (*R* = 0.64, *P* < 0.001). Deming regression showed an intercept of −0.31 (95% CI: −2.38 to 1.76) and a slope of 0.99 (95% CI: 0.88–1.09) as shown in *Figure 2A*, indicating no significant constant or proportional bias of GLS measurements between the two methods. The agreement between AI and expert measurements was moderate (ICC = 0.63, 95% CI: 0.56–0.69. *P* < 0.001), which did not meet the pre-specified exploratory benchmark of 0.70. The mixed-effects Bland–Altman analysis that included random intercepts for individual subjects demonstrated a mean measurement bias of 0.55%, and no statistically significant evidence of proportional measurement bias (β = 0.033, *P* = 0.72), with limits of agreement of −3.63% to 4.73% (*Figure 2B*).

### Temporal changes in GLS

**Figure 3:**
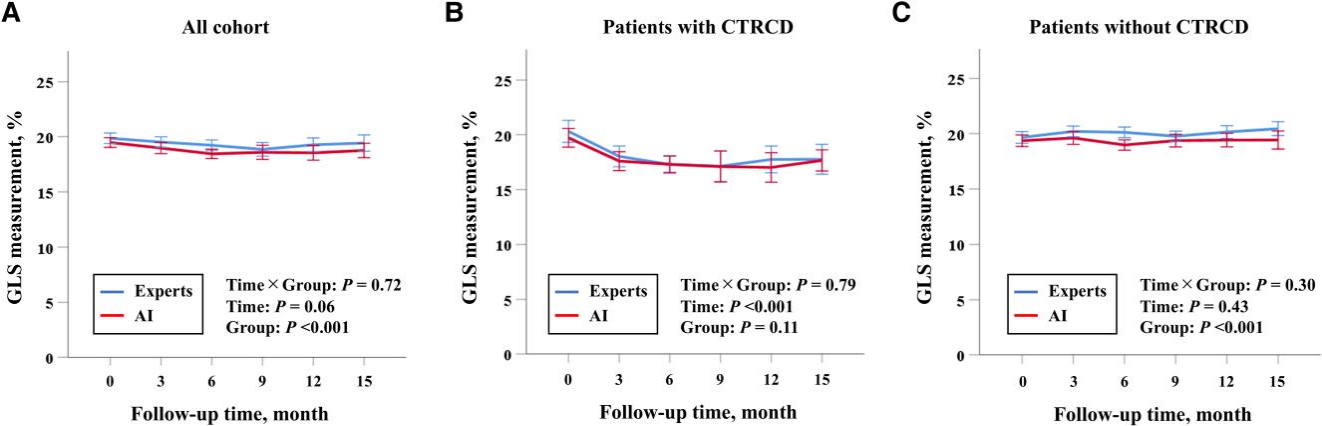
Longitudinal changes in GLS measured by experts and AI. Estimated marginal means of GLS derived from linear mixed-effects models are plotted for each group. Blue lines: expert measurements; red lines: AI-based measurements. Panels: (*A*) overall cohort, (*B*) GLS-derived CTRCD subgroup, (*C*) non-CTRCD group. AI, artificial intelligence; CTRCD, cancer therapy-related cardiac dysfunction; GLS, global longitudinal strain.

To assess the differences in longitudinal changes in GLS between AI and experts, the linear mixed-effects model analyses were performed as shown in *Figure 3*. The time × group (experts vs. AI) interaction was not statistically significant (*P* = 0.72, *Figure 3A*), indicating similar temporal GLS trajectories between the two methods. The main effect of time was not significant (*P* = 0.060), suggesting no overall change of GLS over time in either group. In contrast, AI-derived GLS values remained consistently lower than expert measurements at all time points (*P* < 0.001). When patients were stratified by the presence or absence of GLS-based CTRCD, no significant difference was observed between the two groups in the time × group interaction (*Figure 3B* and *C*).

### Detection of CTRCD

**Figure 4:**
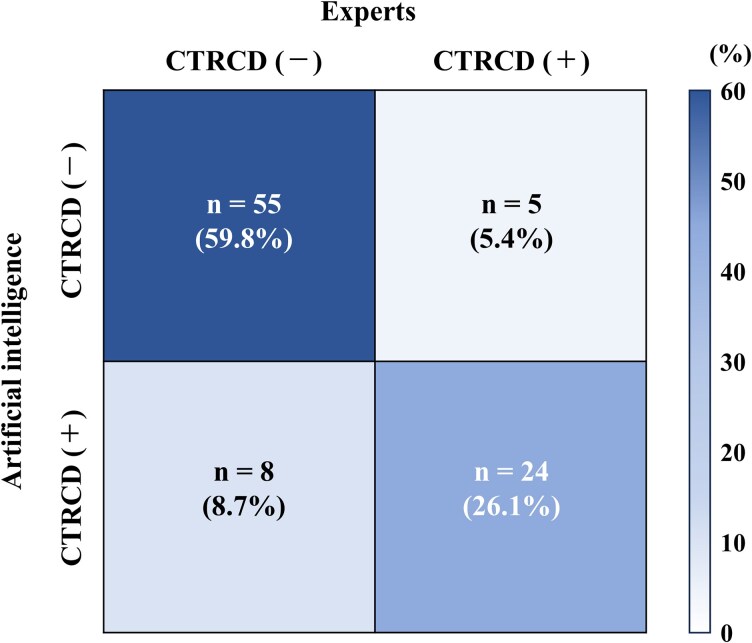
Confusion matrix comparing CTRCD classification. The heatmap illustrates the agreement between the fully automated AI-based echocardiographic analysis and expert manual assessment in classifying CTRCD. AI, artificial intelligence; CTRCD, cancer therapy-related cardiac dysfunction; GLS, global longitudinal strain.

Among 92 patients, 29 patients (31.5%) developed GLS-based CTRCD during follow-up on expert measurements, and 32 patients (34.8%) on AI-derived measurements. The detection rates of GLS-based CTRCD were similar between AI and experts, with no statistically significant difference (*P* = 0.58). There was no significant difference in the timing of GLS-based CTRCD detection between AI and expert assessments on the Wilcoxon signed-rank test (median detection time: 9.1 months [IQR, 6.0–12.3 months] vs. 9.0 months [IQR, 5.9–12.0 months]; *P* = 0.47). The presence or absence of GLS-based CTRCD showed an 85.9% concordance between the two methods, and agreement for GLS-based CTRCD detection was substantial (Cohen’s kappa coefficient = 0.68, 95% CI = 0.60–0.76, *P* < 0.001). In addition, a detailed comparison of classification performance is shown in *Figure 4*. An AI-based system reproduced expert assessment with good accuracy. Sensitivity and specificity for detecting GLS-based CTRCD were 82.8% and 87.3%, respectively, with a positive predictive value of 75%, and a negative predictive value of 91.7%, when expert assessment was used as the reference standard.

Only three patients met expert-derived LVEF-based CTRCD criteria during follow-up. Therefore, comparing the incidence of LVEF-based CTRCD according to the presence or absence of GLS-based CTRCD was not feasible. However, patients classified as having GLS-based CTRCD by AI exhibited significantly greater LVEF decline during follow-up compared with those without CTRCD (ΔLVEF: 10.7 ± 10.4% vs. 5.1 ± 6.5%, *P* = 0.008).

### Reproducibility of manual GLS measurements

To assess intra-observer and inter-observer variability, two independent experts performed an analysis of GLS for 20 patients on two different occasions. Intra-observer variability was expressed as the standard error of measurement (SEM_intra_), and inter-observer variability as SEM_inter_.^15^ All measurements were made by each observer, blinded to previous measurements and to the measurements of the other observer. For GLS measurements, SEM_intra_ and SEM_inter_ were 0.8% and 1.1%, respectively.

## Discussion

In this prospective study, we investigated the diagnostic performance of a fully automated AI-based echocardiographic analysis system for monitoring temporal GLS changes and detecting GLS-based CTRCD in breast cancer patients undergoing cardiotoxic chemotherapy. The main findings of the present study are as follows: (1) AI-derived GLS values were slightly but significantly lower than expert-derived values; nevertheless, the two methods showed a moderate correlation and agreement of GLS measurements; (2) temporal GLS trajectories during and after chemotherapy were highly concordant between the two methods; and (3) the AI-based system demonstrated substantial concordance with experts in detecting GLS-based CTRCD, with similar detection rates and timing.

The importance of GLS monitoring in cardio-oncology is emphasized in contemporary guidelines, as GLS reduction precedes overt LVEF decline and enables early initiation of cardioprotective strategies.^1,16,17^ However, widespread implementation of GLS measurements into routine echocardiographic practice remains limited due to technical demands, susceptibility to inter-observer variability, and the additional time required for high-quality strain analysis.^18,19^ The system may be especially beneficial in institutions with limited expert availability or high clinical workload. The present findings support the potential role of fully automated AI-based echocardiographic analysis as a supportive tool for serial GLS surveillance. However, the observed level of agreement does not support unrestricted replacement of expert GLS analysis at the individual-study level.

Although absolute AI-derived GLS values were lower than expert measurements, this systematic offset did not translate into proportional bias as demonstrated by Deming regression and mixed-effects Bland–Altman analysis. A potential explanation for the systematic difference in absolute GLS values is the difference in strain analysis algorithms between a fully automated AI system (Us2.ai) and the vendor-specific software used for expert measurements (EchoPAC). The underestimation also likely reflects more consistent and inward contouring by the AI system, a pattern also reported in previous automated strain studies.^20^ The observed ICC of 0.63 for GLS measurements between AI and experts did not meet the pre-specified exploratory target of 0.70. Several previous studies have demonstrated a similar level of agreement, corresponding to the moderate reproducibility of manual GLS analysis across different vendors and software platforms, with ICC values typically ranging from 0.55 to 0.78.^21,22^ Because both AI and expert analyses were performed on the same General Electric Company-acquired image set, a higher level of agreement would ideally be expected. Therefore, these findings should not be interpreted as evidence of full interchangeability between methods.

CTRCD diagnosis based on GLS relies on relative change rather than absolute values. Thus, the system’s ability to reproduce temporal GLS trajectories is crucial. The present study focused on longitudinal comparisons of serial GLS measurements, unlike previous studies assessing primarily cross-sectional agreement between AI- and expert-derived echocardiographic parameters. Evaluating temporal GLS trajectories rather than one-point agreement provides deeper insight into whether AI can faithfully reproduce dynamic changes in cardiac function observed during and after chemotherapy. The present study demonstrated consistent temporal GLS trends between AI and expert measurements, supporting the feasibility of using AI for serial GLS monitoring in cardio-oncology practice. The diagnostic performance metrics further support the clinical utility of the automated system. With a sensitivity of 82.8% and a specificity of 87.3%, along with a high negative predictive value of 91.7%, the AI system reliably excluded CTRCD in most patients. These characteristics are consistent with its proposed role as a supportive tool for longitudinal surveillance, where ruling out early cardiotoxicity is clinically important. Furthermore, patients identified as having GLS-based CTRCD by the AI system demonstrated a significantly greater subsequent decline in LVEF. However, because only three patients met LVEF-based CTRCD criteria, this finding should be interpreted cautiously and considered exploratory and hypothesis-generating rather than definitive evidence of prognostic utility.

AI-derived GLS should not yet replace expert interpretation in all clinical scenarios. The within-subject variability observed in Bland–Altman analysis indicates that single-time-point interchangeability between experts and AI remains imperfect. Caution may be warranted when interpreting single, isolated AI-derived GLS values, especially in borderline cases of GLS-based CTRCD. Because GLS-based CTRCD is defined by a relative reduction from baseline, systematic underestimation of the absolute baseline GLS value may mathematically affect the calculated percentage change and contribute to discordant threshold-based CTRCD classification, particularly in borderline cases near the >15% cut-off. To further explore this issue, we additionally examined relative GLS changes from baseline and found that longitudinal changes were broadly similar between methods despite the systematic offset in absolute values, with no significant time-by-group interaction (see Supplementary material online, *Figure S1*). Expert review and integration with other clinical data, such as symptoms, biomarkers, and other imaging data, remain essential in contemporary cardio-oncology surveillance, when values fluctuate abruptly or when clinical decisions carry significant implications like modifying chemotherapy regimens.

## Limitations

Our study has several limitations. First, this was a single-centre study with modest sample size and specific vendors, which may limit generalizability. The study was primarily designed for the evaluation of measurement agreement and longitudinal tracking performance, rather than for detecting small differences in CTRCD incidence or timing between methods. The present study may have been underpowered to detect very small differences in CTRCD detection rates between AI and expert measurements. At the same time, we recognize that the present study did not include a formal pre-specified qualitative adjudication of all discordant cases. Second, the gold standard used for comparison was manual expert analysis, which is not without its own variability, but is widely accepted. Unsuccessful AI-derived GLS measurements represent a practical limitation of automated analysis, particularly in studies with suboptimal apical views and/or insufficient endocardial visualization. Understanding such failure patterns is essential for clinical implementation and should be addressed more systematically in future multi-centre studies. Third, biomarkers such as serum troponin were not incorporated into the diagnosis of CTRCD in the present study.^23^ Fourth, findings from the present study should be interpreted as platform-specific and should not be generalized to other vendors or AI tools without caution. The present findings should not be extrapolated to other vendors or acquisition environments without external validation. Fifth, a few patients developed LVEF-based CTRCD, limiting the assessment of whether GLS-based CTRCD detected by AI predicts subsequent clinical systolic dysfunction or cardiac events. However, exploratory analysis demonstrated that AI-derived GLS-based CTRCD was associated with greater subsequent LVEF decline, supporting its potential prognostic value. Larger multi-centre studies across multiple vendors are needed to validate AI-derived GLS for routine clinical decision-making and to determine whether AI-identified GLS deterioration predicts future LVEF decline and long-term cardiac outcomes.

## Conclusions

In this prospective longitudinal study, the fully automated echocardiographic analysis system underestimated absolute GLS values and did not achieve the pre-specified agreement target for full interchangeability with expert measurements. However, it reproduced longitudinal GLS trajectories reasonably well and showed good overall concordance for GLS-based CTRCD classification. These findings suggest that AI-based automated GLS analysis may serve as a supportive tool for serial cardio-oncology surveillance, while expert interpretation remains essential in some cases.

## Supplementary Material

ztag097_Supplementary_Data

## Date availability

The data underlying this article are available within the article itself and in the accompanying online supplementary material.

## References

[1] Lyon AR, López-Fernández T, Couch LS, Asteggiano R, Aznar MC, Bergler-Klein J, et al 2022 ESC guidelines on cardio-oncology developed in collaboration with the European Hematology Association (EHA), the European Society for Therapeutic Radiology and Oncology (ESTRO) and the International Cardio-Oncology Society (IC-OS). Eur Heart J 2022;43:4229–4361. 36017568 10.1093/eurheartj/ehac244

[2] Okushi Y, Saijo Y, Yamada H, Toba H, Zheng R, Seno H, et al Effectiveness of surveillance by echocardiography for cancer therapeutics-related cardiac dysfunction of patients with breast cancer. J Cardiol 2023;82:467–472. 37481235 10.1016/j.jjcc.2023.07.002

[3] Saijo Y, Kusunose K, Yamada N, Yamada H, Nishio S, Hirata Y, et al Sequential speckle tracking imaging to detect early stage of cancer therapeutics-related cardiac dysfunction in a patient with breast cancer. J Echocardiogr 2020;18:134–135. 30810909 10.1007/s12574-019-00423-2

[4] Thavendiranathan P, Negishi T, Somerset E, Negishi K, Penicka M, Lemieux J, et al Strain-guided management of potentially cardiotoxic cancer therapy. J Am Coll Cardiol 2021;77:392–401. 33220426 10.1016/j.jacc.2020.11.020

[5] Yu CM . Challenges and opportunity in the era of quantitative echocardiography. Echo Res Pract 2017;4:E3–eE6. 28899862 10.1530/ERP-17-0049 PMC5633054

[6] Negishi K, Negishi T, Kurosawa K, Hristova K, Popescu BA, Vinereanu D, et al Practical guidance in echocardiographic assessment of global longitudinal strain. JACC Cardiovasc Imaging 2015;8:489–492. 25129519 10.1016/j.jcmg.2014.06.013

[7] Liu JE, Barac A, Thavendiranathan P, Scherrer-Crosbie M. Strain imaging in cardio-oncology. JACC CardioOncol 2020;2:677–689. 34396282 10.1016/j.jaccao.2020.10.011 PMC8352045

[8] Tromp J, Bauer D, Claggett BL, Frost M, Iversen MB, Prasad N, et al A formal validation of a deep learning-based automated workflow for the interpretation of the echocardiogram. Nat Commun 2022;13:6776. 36351912 10.1038/s41467-022-34245-1 PMC9646849

[9] Hirata Y, Nomura Y, Saijo Y, Sata M, Kusunose K. Reducing echocardiographic examination time through routine use of fully automated software: a comparative study of measurement and report creation time. J Echocardiogr 2024;22:162–170. 38308797 10.1007/s12574-023-00636-6 PMC11343801

[10] Koo TK, Li MY. A guideline of selecting and reporting intraclass correlation coefficients for reliability research. J Chiropr Med 2016;15:155–163. 27330520 10.1016/j.jcm.2016.02.012 PMC4913118

[11] Taub CC, Stainback RF, Abraham T, Forsha D, Garcia-Sayan E, Hill JC, et al Guidelines for the standardization of adult echocardiography reporting: recommendations from the American society of echocardiography. J Am Soc Echocardiogr 2025;38:735–774. 40912865 10.1016/j.echo.2025.06.001

[12] Mitchell C, Rahko PS, Blauwet LA, Canaday B, Finstuen JA, Foster MC, et al Guidelines for performing a comprehensive transthoracic echocardiographic examination in adults: recommendations from the American society of echocardiography. J Am Soc Echocardiogr 2019;32:1–64. 30282592 10.1016/j.echo.2018.06.004

[13] Bonett DG . Sample size requirements for estimating intraclass correlations with desired precision. Stat Med 2002;21:1331–1335. 12111881 10.1002/sim.1108

[14] Parker RA, Scott C, Inácio V, Stevens NT. Using multiple agreement methods for continuous repeated measures data: a tutorial for practitioners. BMC Med Res Methodol 2020;20:154. 32532218 10.1186/s12874-020-01022-x PMC7291585

[15] Eliasziw M, Young SL, Woodbury MG, Fryday-Field K. Statistical methodology for the concurrent assessment of interrater and intrarater reliability: using goniometric measurements as an example. Phys Ther 1994;74:777–788. 8047565 10.1093/ptj/74.8.777

[16] Negishi K, Negishi T, Haluska BA, Hare JL, Plana JC, Marwick TH. Use of speckle strain to assess left ventricular responses to cardiotoxic chemotherapy and cardioprotection. Eur Heart J Cardiovasc Imaging 2014;15:324–331. 24057661 10.1093/ehjci/jet159

[17] Negishi T, Thavendiranathan P, Penicka M, Lemieux J, Murbraech K, Miyazaki S, et al Cardioprotection using strain-guided management of potentially cardiotoxic cancer therapy: 3-year results of the SUCCOUR trial. JACC Cardiovasc Imaging 2023;16:269–278. 36435732 10.1016/j.jcmg.2022.10.010

[18] Gong F, Akhter N, Vaitenas I, Wodzinski B, Lancki N, Welty LJ, et al Reliability of left ventricular global longitudinal strain across acquisition and analysis techniques: a prospective comparative study. Eur Heart J Imaging Methods Pract 2025;3:qyaf101. 40909131 10.1093/ehjimp/qyaf101 PMC12405870

[19] Negishi T, Negishi K, Thavendiranathan P, Cho GY, Popescu BA, Vinereanu D, et al Effect of experience and training on the concordance and precision of strain measurements. JACC Cardiovasc Imaging 2017;10:518–522. 27743951 10.1016/j.jcmg.2016.06.012

[20] Lafitte S, Lafitte L, Jonveaux M, Pascual Z, Ternacle J, Dijos M, et al Integrating artificial intelligence into an echocardiography department: feasibility and comparative study of automated versus human measurements in a high-volume clinical setting. Arch Cardiovasc Dis 2025;118:477–488. 40340211 10.1016/j.acvd.2025.04.051

[21] Farsalinos KE, Daraban AM, Ünlü S, Thomas JD, Badano LP, Voigt JU. Head-to-head comparison of global longitudinal strain measurements among nine different vendors: the EACVI/ASE inter-vendor comparison study. J Am Soc Echocardiogr 2015;28:1171–81, e2. 26209911 10.1016/j.echo.2015.06.011

[22] Nagata Y, Takeuchi M, Mizukoshi K, Wu VC, Lin FC, Negishi K, et al Intervendor variability of two-dimensional strain using vendor-specific and vendor-independent software. J Am Soc Echocardiogr 2015;28:630–641. 25747915 10.1016/j.echo.2015.01.021

[23] Loganath K, Lee KK, Oikonomidou O, Hall P, Mills NL, Joshi S, et al Anthracycline dose, myocardial injury, and change in left ventricular function in the cardiac CARE trial. JACC CardioOncol 2025;7:725–735. 40810716 10.1016/j.jaccao.2025.06.003 PMC12790034

