# Supplementary material for: Fully automated artificial intelligence–based echocardiographic analysis for global longitudinal strain monitoring and cancer therapy–related cardiac dysfunction detection in breast cancer patients
Source: Eur Heart J Digit Health. 2026 Jun 19;7(6):ztag097. doi: 10.1093/ehjdh/ztag097 (PMC13322393; doi:10.1093/ehjdh/ztag097)
Supplement: ztag097_Supplementary_Data [file ztag097_supplementary_data.docx]

**Figure S1 Relative GLS changes from baseline**


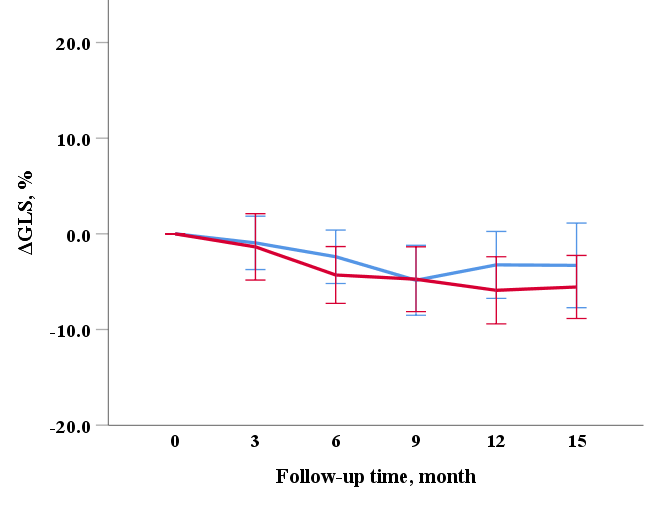


**AI**

**Experts**

**Time×Group: *P* = 0.30**

**Time: *P* <0.001**

**Group: *P* = 0.80**

Longitudinal changes in GLS were broadly similar between two methods despite systematic offset in absolute values. The shaded area represents the normal range.

**Table S1 Characteristics of patients with discordant GLS-based CTRCD classification between experts and AI assessment**

| **ID** | **Baseline GLS (Expert, %)** | **Baseline GLS (AI, %)** | **Max Relative GLS Reduction (Expert, %)** | **Max Relative GLS Reduction (AI, %)** | **Discordance Type** | **Visit Number of Expert-defined CTRCD** | **Visit Number of AI-defined CTRCD** |
| --- | --- | --- | --- | --- | --- | --- | --- |
| 1 | 22.7 | 21.6 | 14.4 | **21** | Expert-/AI+ | - | 1 |
| 10 | 20.5 | 19.6 | **20.5** | 9.7 | Expert+/AI- | 5 | - |
| 20 | 12.6 | 14.8 | **21.4** | 8.8 | Expert+/AI- | 1 | - |
| 35 | 18 | 17.8 | **17.8** | 2.2 | Expert+/AI- | 3 | - |
| 38 | 18.4 | 19.7 | 9.8 | **20.8** | Expert-/AI+ | - | 4 |
| 42 | 20.5 | 19.8 | **16.7** | -3 | Expert+/AI- | 1 | - |
| 56 | 21.8 | 22.1 | 11.5 | **28.8** | Expert-/AI+ | - | 4 |
| 62 | 17.1 | 19.5 | 14 | **16.4** | Expert-/AI+ | - | 2 |
| 67 | 18.9 | 20.6 | 8.5 | **18.5** | Expert-/AI+ | - | 4 |
| 77 | 21.2 | 20.3 | 12.7 | **22.3** | Expert-/AI+ | - | 1 |
| 82 | 20.9 | 21 | 11.5 | **21.4** | Expert-/AI+ | - | 3 |
| 91 | 19.7 | 21.6 | **21.8** | 10.2 | Expert+/AI- | 1 | - |
| 92 | 18.3 | 20.5 | 6 | **27.3** | Expert-/AI+ | - | 2 |

CTRCD was defined as a >15% relative reduction in GLS from baseline. Discordant cases were defined as patients classified differently by expert and AI assessment at any time during follow-up. Visit numbers indicate the first follow-up visit at which CTRCD criteria were met by each method. A dash indicates that CTRCD criteria were not met by that method during follow-up. Negative values indicate an apparent increase in absolute GLS relative to baseline.
